# Multicenter Evaluation of the Novel ETEST Fosfomycin for Antimicrobial Susceptibility Testing of Enterobacterales, Enterococcus faecalis, and Staphylococcus Species

**DOI:** 10.1128/jcm.00021-22

**Published:** 2022-06-23

**Authors:** A. Goer, L. S. Blanchard, A. Van Belkum, K. J. Loftus, T. P. Armstrong, S. G. Gatermann, D. Shortridge, B. J. Olson, J. K. Meece, T. R. Fritsche, M. Pompilio, D. Halimi, C. Franceschi

**Affiliations:** a Institut fuer Medizinische Laboratiums Diagnostik & National Reference Centre for Multidrug-Resistant Gram-Negative Bacteria, Department of Medical Microbiology, Ruhr-University, Bochum, Germany; b bioMérieux Global Clinical Affairs, Marcy-L’Étoile, France; c bioMérieux, La Balme les Grottes, France; d bioMérieux Global Clinical Affairs, Hazelwood, Missouri, USA; e JMI Laboratories, North Liberty, Iowa, USA; f Marshfield Clinic Health System, Marshfield, Wisconsin, USA; Johns Hopkins

**Keywords:** antimicrobial susceptibility testing, ETEST, Enterobacterales, Enterococcus faecalis, fosfomycin, *Staphylococcus* spp., gradient methods

## Abstract

Fosfomycin is a phosphonic acid derivative active against a wide spectrum of Gram-positive and Gram-negative pathogens. It is used for the treatment of uncomplicated urinary tract infections (uUTI) or severe infections by oral or intravenous (i.v.) administration. In order to improve its performance and robustness, the fosfomycin strip, an antibiotic gradient diffusion strip, was redeveloped and evaluated in the multicenter study summarized in this paper. ETEST fosfomycin (ETEST FO) clinical performance was evaluated by three study sites on 152 Enterococcus faecalis, 100 Staphylococcus spp. and 330 Enterobacterales in comparison with the CLSI and EUCAST agar dilution reference method. Referring to FDA performance criteria, the ETEST FO achieved 91.0% of essential (EA) and 99.0% of categorical agreement (CA) for Escherichia coli. In addition, 98.0% EA and 93.4% CA were achieved for E. faecalis, with no very major errors (VME) or major errors (ME). According to EUCAST breakpoints for intravenous fosfomycin use, Enterobacterales and Staphylococcus spp. also met ISO acceptance criteria for EA and CA (EA 91.5%, 94.0%, respectively, and CA 98.0% for both). A VME rate of 8.8% was observed for Enterobacterales but the MICs were within EA. A trend to predict lower MICs for Citrobacter spp., E. coli and Salmonella enterica and to predict higher MICs for Klebsiella pneumoniae MICs was observed, while ETEST FO should not be used for Enterobacter cloacae, because of low EA and a high VME rate. The study results support the efficiency of the novel ETEST FO, making it an easy-to-handle tool as a substitute to the classical agar dilution method.

## INTRODUCTION

Fosfomycin (FO) is a bactericidal antibiotic that disrupts cell wall synthesis by inhibiting phosphoenolpyruvate synthetase thus interfering with the production of peptidoglycan. FO shows *in vitro* activity against a broad spectrum of Gram-positive and Gram-negative bacteria. The phosphonic acid derivative was first discovered in 1969 (1), and is available as fosfomycin trometamol in the United States and Europe (EU) for oral use, as well as fosfomycin (di-)sodium for intravenous application in most EU countries (2, 3). It is excreted in unchanged form in urine and feces; hence, qualifying for the treatment of urinary tract infections. Fosfomycin trometamol was approved by the U.S. Food and Drug Administration (FDA) for treatment of uncomplicated urinary tract infections (uUTI) in women caused by susceptible strains of Escherichia coli and Enterococcus faecalis (2). The European Medicines Agency (EMA) approved fosfomycin trometamol for oral use in uUTI in adult and adolescent females and for intravenous use for all susceptible pathogens. EMA has recently made recommendations to only use intravenous FO for serious or complicated infections when other antibiotic treatments are not feasible (3). The European Committee on Antimicrobial Susceptibility Testing (EUCAST) provide different FO breakpoints, one for oral treatment of uUTI for E. coli and a different breakpoint for FO i.v. use for Enterobacterales and Staphylococcus spp. (4–6). EUCAST also recommends use of FO in severe infections always in combination with a β-lactam, aminoglycoside, fluoroquinolone, or glycopeptide antibiotic (4). FO has been long established for treating UTI, but due to the emergence of multidrug resistant pathogens (MDR) FO caught attention because there is no cross-resistance known to pathogens producing extended-spectrum-beta-lactamase (ESBL) or carbapenemase-producing Enterobacterales, vancomycin-resistant enterococci (VRE), or methicillin-resistant Staphylococcus aureus (MRSA). Studies showed that FO can be used among others as an additive and synergistic compound against Gram-positive or Gram-negative MDR, and also has favorable pharmacokinetics (for example, penetrating biofilms and abscesses) making it an interesting tool for modern anti-infective therapy (7–13).

Unfortunately, the antimicrobial susceptibility testing (AST) for FO is challenging. EUCAST and CLSI both recommended agar dilution (AD) as the reference method. Disk diffusion can be used, but only for E. coli, and CLSI even restricts it further to only E. coli from urine samples. For the diagnostic laboratory, a fast and easy to perform testing method for determining the MIC for Gram-positive and Gram-negative isolates is needed. The ETEST based on a combination of dilution and diffusion testing offers a valuable solution. However, publications about ETEST FM, the previous Fosfomycin strip, showed concerns about the accuracy of results. One aspect was that micro- and macrocolonies lead to misinterpretation and incorrectly read MIC values with poor EA and high VME rates, especially for Klebsiella spp. (14–18).

The purpose of the present multicenter trial was to demonstrate the optimized performance of the newly developed ETEST FO (bioMérieux, Marcy l’Etoile, France) in determining the MIC of Enterobacterales, E. faecalis, and Staphylococcus spp. compared with the AD reference method.

## MATERIALS AND METHODS

### Setting.

The purpose of our study was to demonstrate the performance of the ETEST FO compared with the Clinical and Laboratory Standards Institute (CLSI) AD reference method as described in M100-Ed31 (2021) and M07-Ed11 (2018) (19, 20).

The clinical trial comprised (i) a quality control study, (ii) a reproducibility study, (iii) a clinical study, and (iv) a challenge study. Three clinical trial sites were included: JMI Laboratories (IA, USA); Marshfield Clinic Health System (WI, USA), and National Reference Centre for Multidrug-Resistant Gram-Negative Bacteria with IML GmbH Bochum (Bochum, Germany).

Performance of ETEST FO was evaluated using EUCAST, CLSI/FDA breakpoints as applicable (Tables 1 and 2).

**TABLE 1:** CLSI/FDA breakpoints for FDA performance

Organisms	Minimum inhibitory concentrations (μg/mL)		
	Susceptible (S)	Intermediate (I)	Resistant (R)
Escherichia coli (Fosfomycin oral and UTI only)	≤64	128	≥256
Enterococcus faecalis (Fosfomycin oral and UTI only)	≤64	128	≥256

**TABLE 2:** EUCAST breakpoints for ISO performance

Organisms	Minimum inhibitory concentrations (μg/mL)	
	Susceptible (S)	Resistant (R)
Enterobacterales (Fosfomycin i.v.)	≤32	>32
Escherichia coli (Fosfomycin oral and uUTI only)	≤8	>8
Staphylococcus spp. (Fosfomycin i.v.)	≤32	>32

### Quality control study.

Three ATCC reference strains for ETEST FO and four ATCC reference strains for AD, as recommended by CLSI M100-Ed31 (2021) (19) and EUCAST v11.0 QC table (4), were tested as quality controls on each day of comparative testing on Mueller-Hinton (Becton, Dickinson and Company; Franklin Lakes, USA). The following ATCC strains were tested with ETEST FO: Enterococcus faecalis ATCC 29212 (range CLSI 32 to 128 μg/mL), Staphylococcus aureus ATCC 29213 (range CLSI/EUCAST 0.5 to 4 μg/mL), Escherichia coli ATCC 25922 (range CLSI/EUCAST 0.5 to 2 μg/mL). Pseudomonas aeruginosa ATCC 27853 (range CLSI/EUCAST 2 to 8 μg/mL) was solely used for AD.

### Reproducibility study.

The reproducibility set was composed of 15 on-scale strains (MICs fell between 0.032 and 512 μg/mL) with five E. coli, five E. faecalis, one E. cloacae, one K. pneumoniae, one K. oxytoca, and two Staphylococcus spp. provided by bioMérieux. All strains were tested in triplicate each day for three separate days. The United States claim (E. coli and E. faecalis) and the EU claim (Enterobacterales and Staphylococcus spp.) were using a separate model of 10 organisms of a pre-established reproducibility set as described in the FDA guidance document (21) and the International Organization for Standardization (ISO) document ISO 20776-2 (22).

For each isolate used in the reproducibility study, a modal value was calculated based on the most frequent result. Best- and worst-case calculations of the reproducibility results were calculated. Best-case calculation was based upon the assumption that off-scale values are within one doubling dilution of the mode, while worst-case calculation was based upon the assumption that off-scale values are more than one doubling dilution from the mode.

### Clinical study.

A total of 582 isolates were included in the clinical study, 306 (52.58%) contemporary clinical isolates (collected within 6 months) and 276 from stock collections (47.42%). Each of the three sites tested at least 50 E. faecalis and 50 E. coli strains isolated from urine samples, including 10 ESBL. The EU site tested an additional 179 Enterobacterales, including 50 E. coli isolated from other sources than urines (among them six ESBL), 25 Enterobacter cloacae, nine Citrobacter koseri, 16 Citrobacter freundii, 30 Klebsiella pneumoniae, nine Klebsiella aerogenes, 10 Klebsiella oxytoca, 20 Salmonella enterica, and 10 Serratia marcescens strains. JMI tested an additional 100 Staphylococcus spp. (70 S. aureus and 30 S. epidermidis).

The contemporary clinical isolates were acquired and identified by MALDI-TOF (BRUKER, Billerica, MA, USA and Vitek MS, bioMérieux) from routine cultures processed in the trial site. No duplicate isolates were included.

For testing of each isolate, a 0.5 McFarland suspension was prepared in 0.85% saline, while for mucoid strains, a 1.0 McFarland suspension was used. Incubation time was16 h to 20 h for Enterobacterales and E. faecalis, and 22 h to 24 h for Staphylococcus spp., at 35 ± 2°C.

**FIG 1:**
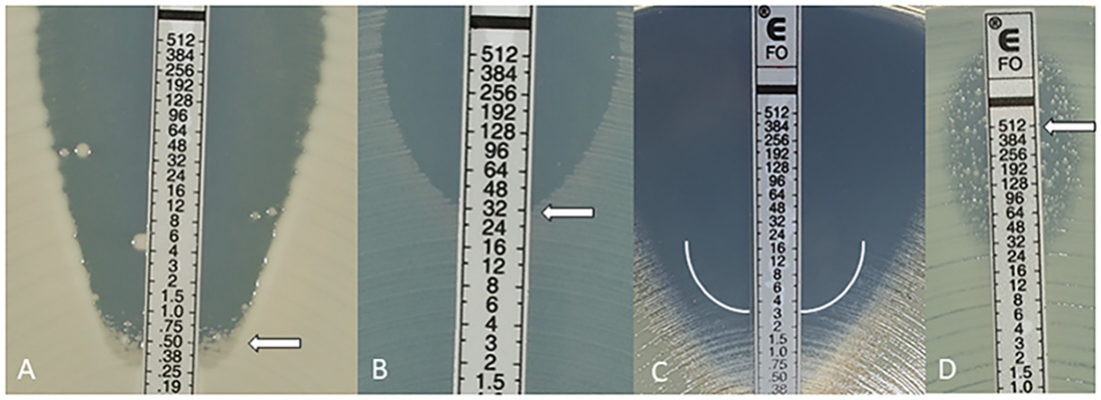
(A) E. coli MIC 0.5 μg/mL: ignore macro- and microcolonies; (B) E. faecalis MIC 32 μg/mL; (C) S. aureus ATCC 29213TM MIC 3 μg/mL: Haze is read as growth; (D) for Enterobacterales, when colonies fill the entire ellipse read ≥512 μg/mL.

Following incubation, a symmetrical inhibition ellipse of microbial growth forms along the strips. The intersection point allows the reading of the MIC value from the scale. For Enterobacterales and E. faecalis, the MIC endpoint was read at 80% of inhibition of growth (bacteriostatic endpoint). Haze-like growth, macro- and microcolonies were ignored, except for colonies filling the entire ellipse, which was considered as MIC ≥ 512 μg/mL (Fig. 1). The MIC endpoint for Staphylococcus spp. is a bactericidal endpoint and therefore it was read at 100% inhibition of growth along the strip. Thin haze as well as macro- and microcolonies, should be considered as growth (Fig. 1).

AD utilizes a set of Mueller-Hinton plates with doubling agar dilution of FO concentrations. Each clinical trial site manufactured the plates using Mueller-Hinton powder, fosfomycin powder, and glucose-6-phosphate powder, provided by bioMérieux. Inoculation was done using a 0.5 McFarland bacterial suspension (also for mucoid strains). The incubation time was16 h to 20 h at 35 ± 2°C. MIC was recorded as the lowest antimicrobial concentration that completely inhibited bacterial growth.

### Challenge study.

JMI Laboratories (IA, USA) tested 76 isolates with 37 E. coli and 39 E. faecalis, including 22 vancomycin-resistant E. faecalis (VRE). E. coli challenge strains expressed a variety of resistance mechanisms: acquired ß-Lactamases, including ESBL and carbapenemases. The resistance mechanisms were identified by whole-genome sequencing for all challenge set strains.

### Data analysis.

ETEST FO results were rounded up to the next AD method dilution when MIC was between two doubling dilutions. Essential agreement (EA) of the ETEST FO results are values that are within a single 2-fold dilution step of the reference method MIC. Categorical agreement (CA) was defined as the percentage of interpretative results (susceptible, intermediate, resistant) in agreement between the ETEST FO and AD.

Discrepancies in interpretation of the MIC values between AD and ETEST FO are defined as minor, major, and very major errors (21, 22).

If the AD result falls within resistant or susceptible interpretative criteria and the ETEST test result is I “intermediate” according to CLSI interpretation, or “susceptible increased exposure” according to EUCAST, and conversely, it is defined as a minor error (mE). A major error (ME) occurs if the AD suggests susceptibility and the ETEST FO result detects resistance and very major error (VME) if the AD result defines resistance and the ETEST result suggests susceptibility.

The interpretative criteria were evaluated by using CLSI and EUCAST breakpoints (Tables 1 and 2). The performance was evaluated based on FDA guidance documents (21) and ISO 20776-2 (22). Both ISO and FDA require EA and CA be equal to or above 90%; ≤3.0% ME; ≥95% reproducibility and quality control within the expected range. FDA approves ≤ 2.0% VME; while ISO requires ≤ 3.0% VME.

## RESULTS

### Quality control study.

For Escherichia coli ATCC 25922, all reference and ETEST FO results were within the expected QC range (100%). One ETEST quality control result was out of range for Enterococcus faecalis ATCC 29212 (68/69), and one ETEST quality control result was out of range for Staphylococcus aureus ATCC 29213 (67/68). The results within expected range were 98.6% for E. faecalis ATCC 29212 and 98.5% for Staphylococcus aureus ATCC 29213 (67/68). In the reference methodology, these ATCC strains were out of range in two cases resulting in 97.1% results within range for both E. faecalis ATCC 29212 (67/69) and S. aureus ATCC 29213 (66/68). For Pseudomonas aeruginosa ATCC 27853, one organism was out of range in AD reference method, resulting in 98.6% results within range. This QC organism is not applicable for ETEST FO given this is not part of the claimed species for ETEST FO. ETEST FO quality control met the FDA and ISO criteria (≥95%) and is acceptable.

### Reproducibility study.

Fifteen isolates were tested in triplicate each day for 3 days at three clinical trial sites with ETEST FO, adding up to a total number of 405 tests. Five E. coli were common to both the United States and EU claim. Both the best- and worst-case calculations of the reproducibility performance achieved 98.1% for the United States claim (265/270), and 97.8% for the EU claim (264/270) (Tables 3 and 4).

**TABLE 3:** Reproducibility performance—United States strains

Organism	No. of results with doubling dilution from the mode							MIC (μg/mL) by test mode
	Off-scale	–2	–1	0	+1	+2	Off-scale	
E. faecalis No. 1				26	1			64
E. faecalis No. 2				18	9			32
E. faecalis No. 3				24	3			32
E. faecalis No. 4				22	5			32
E. faecalis No. 5				27				32
E. coli No. 1				24	3			0.5
E. coli No. 2			11	16				1
E. coli No. 3			12	15				1
E. coli No. 4			11	16				1
E. coli No. 5		5	4	15	3			0.5
Total	0	5	38	203	24	0	0	

**TABLE 4:** Reproducibility performance—EUCAST/ISO strains

Organism	No. of results with doubling dilution from the mode							MIC (μg/mL) by test mode
	Off-scale	–2	–1	0	+1	+2	Off-scale	
E. cloacae			2	25				0.25
E. coli No. 1				24	3			0.5
E. coli No. 2			11	16				1
E. coli No. 3			12	15				1
E. coli No. 4			11	16				1
E. coli No. 5		5	4	15	3			0.5
K. oxytoca				27				4
K. pneumoniae			6	14	7			4
S. epidermidis		1	9	17				2
*S. saprophyticus*				16	11			32
Total	0	6	55	185	24	0	0	

### Challenge study.

For 76 isolates in the challenge study, including E. faecalis (*n* = 39) and E. coli (*n* = 37), the overall EA was 93.4% (71/76) with an EA of 89.2% (33/37) for E. coli and 97.4% (38/39) for E. faecalis. The E. coli isolates that were not in EA had a MIC two to three dilutions lower than the reference results, ranging from 0.064 to 0.5 versus 0.5 to 4 μg/mL, but still fell into CA. The E. faecalis isolate that was not in EA had a MIC two dilutions higher than the reference result, leading to a ME.

The overall CA was 97.4% (74/76), with 94.9% (37/39) and 100% (37/37) for E. faecalis and E. coli, respectively. The VME rate for E. faecalis could not be calculated, because there were no resistant strains available at the time of testing. Among the E. faecalis results were one ME (2.6% 1/38) and one mE (2.6% 1/39). The ME isolate showed a MIC of 256 μg/mL by ETEST FO and a MIC of 64 μg/mL by AD. When analyzing the performance criteria with EUCAST i.v. breakpoints for E. coli, the results were the same. Four FO resistant E. coli isolates were included in the challenge substudy. It may be noted that among the challenge strains, there were 22 VRE, but none of these vancomycin resistant strains were categorized resistant to fosfomycin. All carbapenemase-expressing E. coli were also categorized susceptible to fosfomycin. The four fosfomycin-resistant E. coli strains expressed an ESBL (Table 5).

**TABLE 5:** Challenge study performance

Organism	CLSI breakpoints reference results				Performance of ETEST FO				
	No. of isolates				No. (%) of isolates				
	Total	S (≤64)	I (128)	R (≥256)	EA (%)	CA (%)	VME	ME	mE (%)
E. coli ^*a*^	37	33	0	4	33 (89.2)	37 (100)	0	0	0
E. faecalis	39	38	1	0	38 (97.4)	37 (94.9)	NA^*b*^	1 (2.6)	1 (2.6)
Overall	76	71	1	4	71 (93.4)	74 (97.4)	0	1 (1.4)	1 (1.3)

a Performance results for E. coli according to EUCAST i.v. (S ≤32 mg/L; R >32 mg/L) and oral (S ≤8 mg/L; R >8 mg/L) breakpoints generate the same values. EUCAST does not offer breakpoints for E. faecalis.

b NA, not available.

### Clinical study. (i) ETEST FO performance with CLSI/FDA breakpoints and FDA requirements.

Comparative analysis included a total of 152 E. faecalis and 201 E. coli at the three study sites. We evaluated the performance using CLSI/FDA breakpoints (Table 1) and FDA acceptance criteria. The performance of E. faecalis showed an EA of 98.0% (149/152) and a CA of 93.4% (142/152). There were 0% VME or ME and 6.6% (10/152) minor errors. Of the E. faecalis clinical isolates, 18 were categorized as intermediate and two (2) isolates were resistant (Table 6). The results met all FDA performance criteria.

**TABLE 6:**
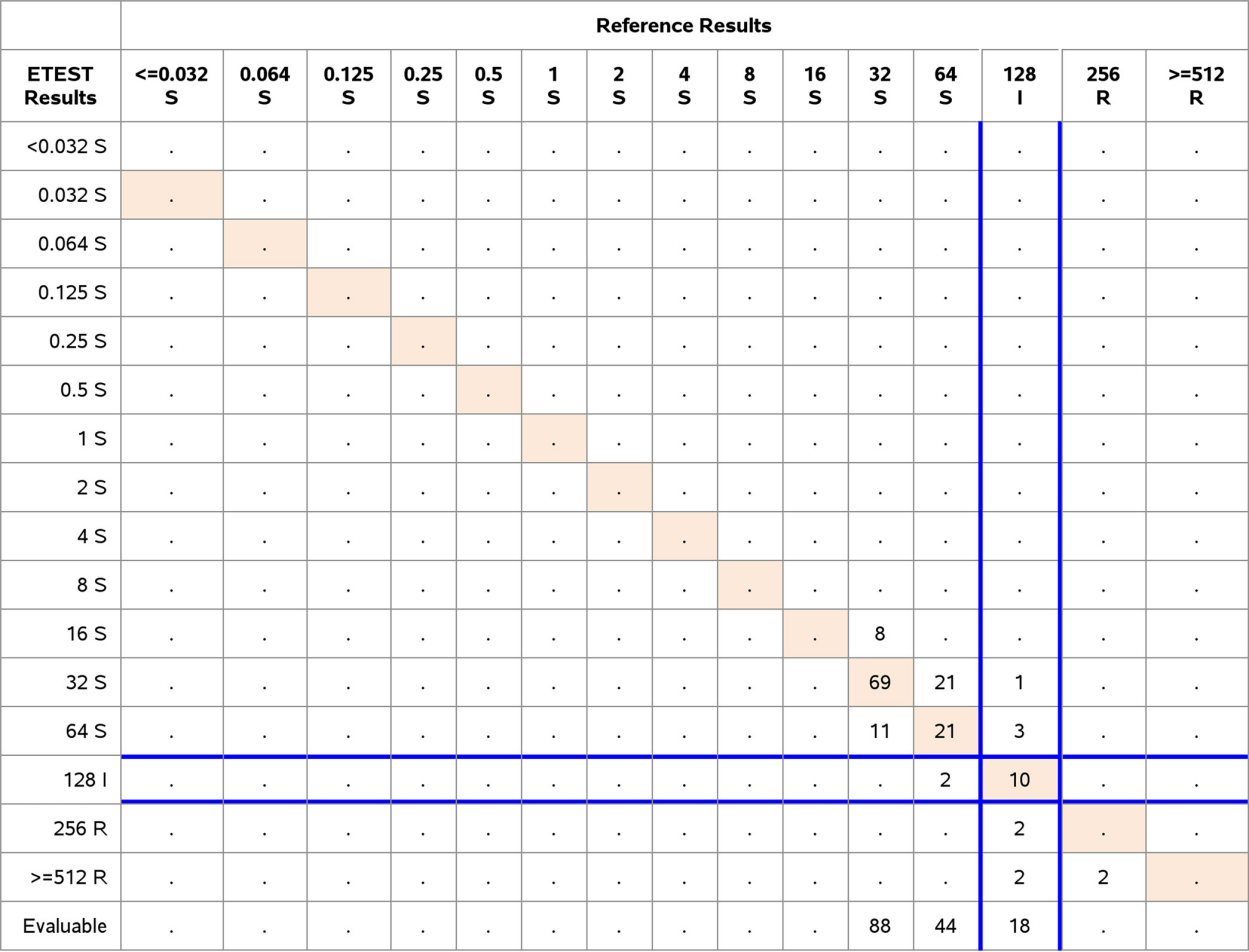
Clinical performance of Enterococcus faecalis, CLSI/FDA breakpoints *n* = 152 (EA 149/152 = 98.0%; CA 142/152 = 93.4%; VME 0/2 resistant isolates = 0%; ME 0/132 susceptible isolates = 0%; mE 10/152 = 6.6%)

The results for E. coli demonstrated 91.0% EA (183/201) and 99.0% CA (199/201), with 0% VME (0/14), 0% ME (0/183) and 1% (2/201) minor errors. According to CLSI/FDA breakpoints, four E. coli strains were categorized intermediate and 14 resistant (Table 7).

**TABLE 7:**
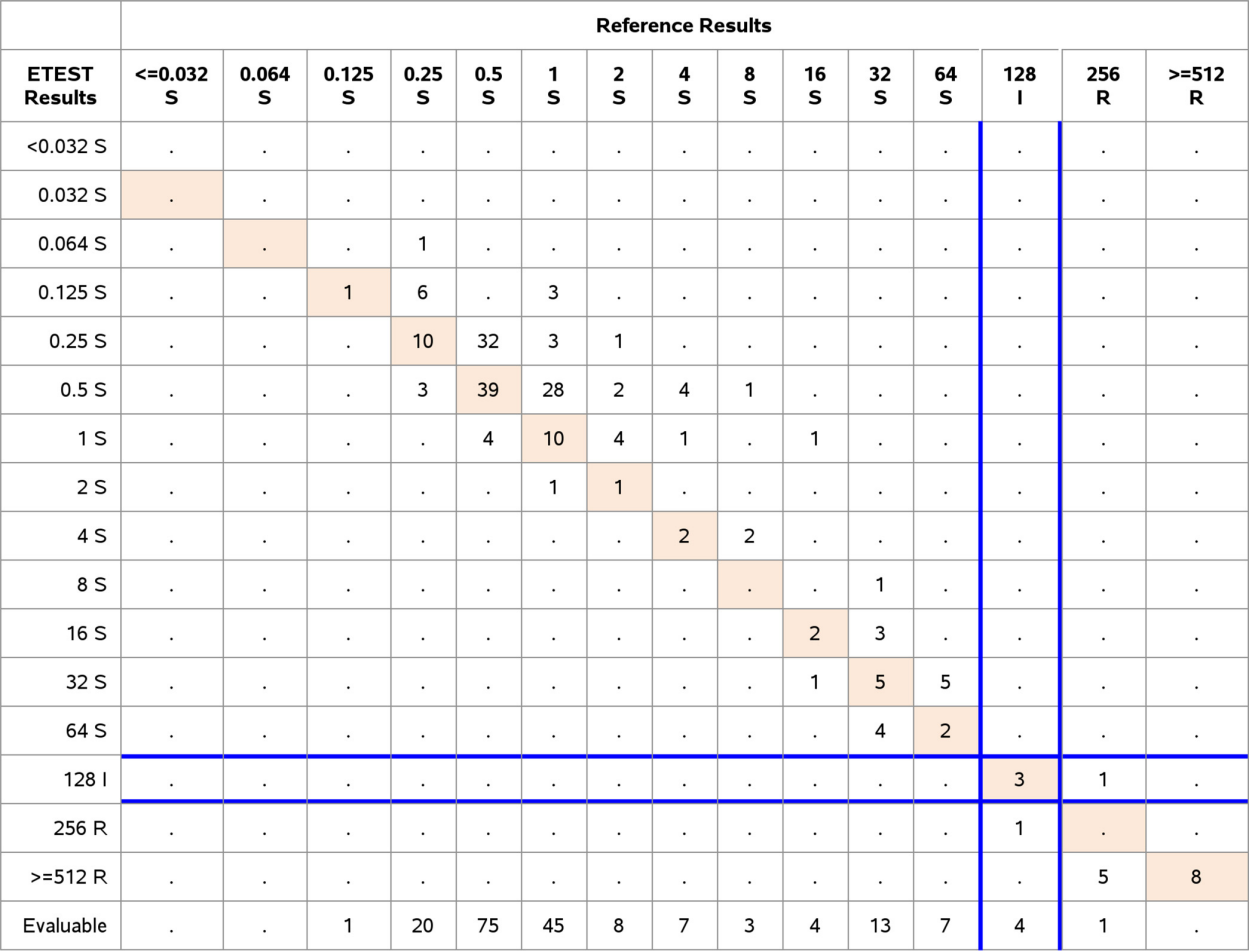
Clinical performance of Escherichia coli, CLSI/FDA breakpoints *n* = 201

**(ii) ETEST FO performance with EUCAST breakpoints and ISO requirements for E. coli and Enterobacterales.** The analysis of performance was based on comparison of the results to the ISO performance criteria. 201 clinical E. coli were analyzed with EUCAST breakpoints for i.v. and oral use. In the former case, an EA and CA was achieved of 91.0% (183/201) and 97.0% (195/201), three ME (1.7%, 3/177) and three VME (12.5%, 3/24) were observed among E. coli isolates. For these VME, the ETEST FO showed a MIC of 32 μg/mL while the AD showed a MIC of 64 μg/mL. The VME were 1 dilution apart from the reference method, consequently they were within EA (Tables 8 and 9). Twenty-four clinical E. coli were categorized resistant with the breakpoint at 32 μg/mL.

**TABLE 8:** Clinical performance results of Enterobacterales and Staphylococcus spp.

Organism	EUCAST i.v. breakpoints reference method			Performance			
	No. (%) of isolates			No. of isolates			
	Total	S (≤32)	R (>32)	EA (%)	CA (%)	VME (%)	ME (%)
C. freundii	16	16	0	15 (93.8)	16 (100)	NA^*a*^	0
*C. koseri*	9	9	0	8 (88.9)	9 (100)	NA	0
E. coli	201	177	24	183 (91.0)	195 (97.0)	3 (12.5)	3 (1.7)
E. coli * (breakpoints oral)	201	160 (≤8)	41 (>8)	183 (91.0)	200 (99.5)	1 (2.4)	0
E. coli breakpoints CLSI	201	183 (≤64)	14 (≤256)	183 (91.0)	199 (99.0)	0	0
K. aerogenes	9	9	0	7 (77.8)	9 (100)	NA	0
K. oxytoca	10	10	0	10 (100)	10 (100)	NA	0
K. pneumoniae	30	20	10	29 (96.7)	30 (100)	0	0
S. enterica	20	20	0	18 (90.0)	20 (100)	NA	0
S. marcescens	10	10	0	9 (90.0)	10 (100)	NA	0
Overall	305	271	34	279 (91.5)	299 (98.0)	3 (8.8)	3 (1.1)
S. aureus	70	63	7	65 (92.9)	69 (98.6)	0	1 (1.6)
S. epidermidis	30	29	1	29 (96.7)	29 (96.7)	0	1 (3.4)
Overall	100	92	8	94 (94.0)	98 (98.0)	0	2 (2.2)

a NA, not available; mE not available.

**TABLE 9:**
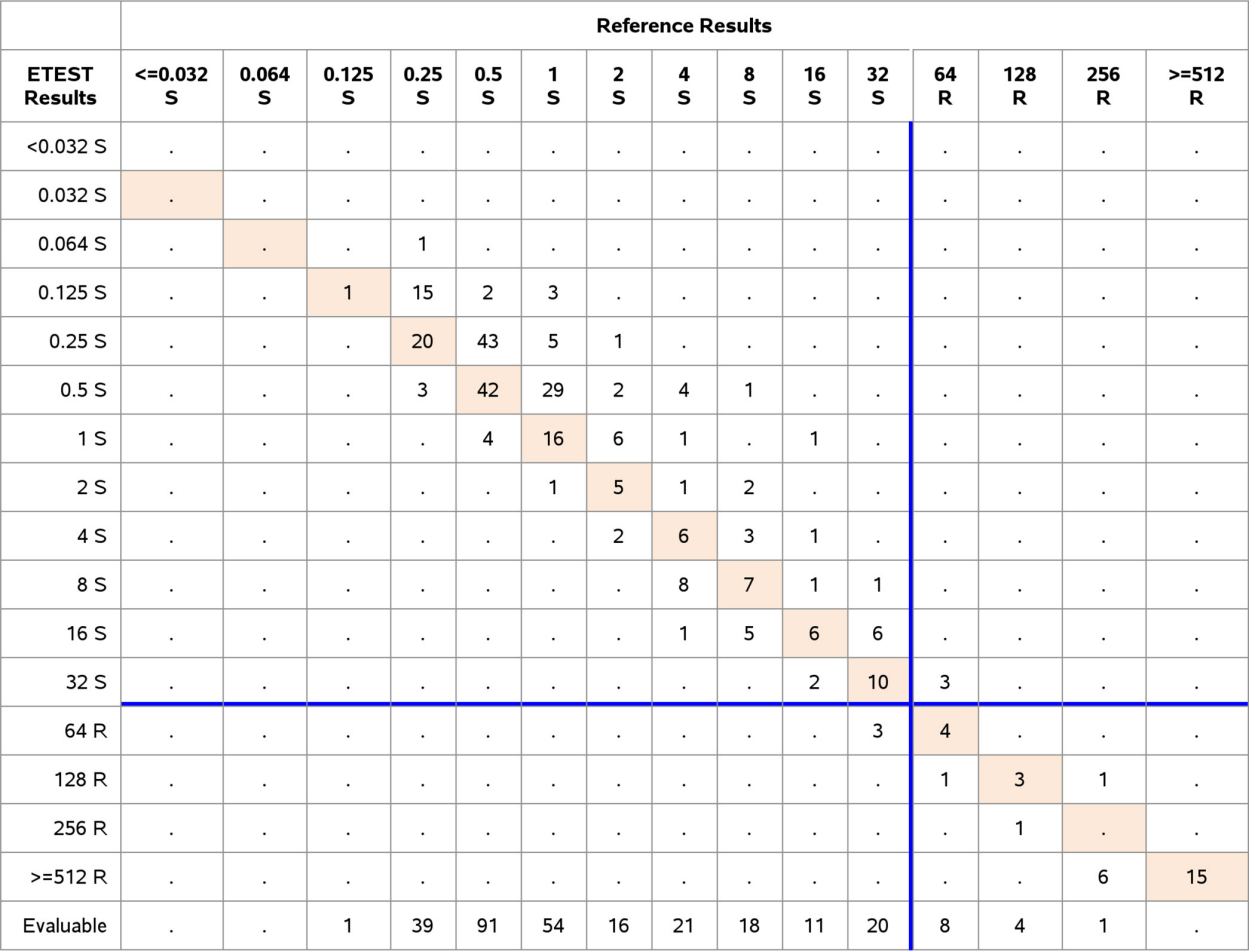
Clinical performance of Enterobacterales, EUCAST i.v. breakpoints *n* = 305^*a*^

a Results exclude E. cloacae.

For oral administration, the overall EA was 91.0% (183/201) and CA was 99.5% (200/201), with 0% ME (0/160) and 2.4% VME (1/41), thus meeting the performance criteria. The breakpoint for oral use is 8 μg/mL, resulting in 41 clinical FO resistant isolates.

Isolates with discrepant VME and ME results were retested according to ISO 20776-2 in triplicate in an effort to resolve the nonmatching results. The presented results are those after resolution. Before resolution, E. coli showed five VME and four ME, after resolution it showed three VME (8.8% 3/34) and ME each (1.1%) (EUCAST i.v. breakpoint) and did not meet VME criteria (≤3.0%). Before resolution E. coli showed two VME and zero ME, after resolution it was one VME and zero ME for the EUCAST oral breakpoint.

For evaluating the performance of Enterobacterales, a total of 330 isolates were tested (including 201 E. coli and 25 E. cloacae). Based on the EUCAST breakpoints for i.v.-administration (S ≤ 32 mg/L, *R* >32 mg/L), the overall EA was 91.5% (279/305) and CA was 98.0% (299/305).

Enterobacter cloacae (*n* = 25) showed unacceptable low EA (88.0%) and CA (92.0%) with two VME (28.6%, seven resistant isolates), even after triplicate testing and were therefore excluded from the study results. Results for Citrobacter spp. showed an EA of 92.0% (23/25) and 100% (25/25) CA, while for Klebsiella spp., an EA of 93.9% (46/49) and a CA 100% (49/49) were determined. The EA for C. koseri and K. aerogenes was low with 88.9% (8/9) and 77.8% (7/9), respectively, although there was a low number of isolates included in the study (each a total of nine). In addition, the other performance criteria for the genus Citrobacter and Klebsiella were fulfilled. Results for S. marcescens presented an EA and CA of 90.0% (9/10) and 100% (10/10), respectively. Data for VME of other Enterobacterales than E. coli and K. pneumoniae is lacking because FO resistant isolates could not be included in the study. There were no ME (0%). The results for each species are shown in Table 8.

**(iii) ETEST FO performance with EUCAST breakpoints and ISO requirements for Staphylococcus spp.** Seventy S. aureus and 30 S. epidermidis were tested according to ISO requirements and EUCAST breakpoint settings (Table 2). The overall CA and EA were 98.0% and 94.0%, respectively, with 2/92 ME (2.2%) and no VME. Only eight resistant isolates could be included in the substudy, with seven resistant S. aureus and one resistant S. epidermidis. In detail, the CA and EA for S. aureus were 98.6% (69/70) and 92.9% (65/70) with one ME (1.6%; 1/63). The CA and EA for S. epidermidis were each 96.7% (29/30) and one ME (3.4%; 1/29) occurred, thus not meeting ISO performance requirements. Both MEs were within EA, because the ETEST showed a MIC of 64 μg/mL and the AD a MIC of 32 μg/mL (Tables 8 and 10).

**TABLE 10:**
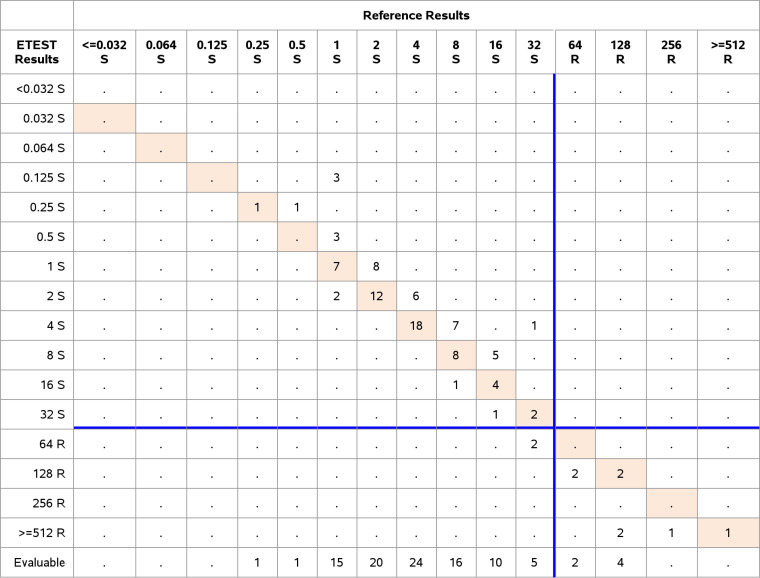
Clinical performance Staphylococcus spp., EUCAST i.v. breakpoints *n* = 100

## DISCUSSION

Fosfomycin is a valuable option for oral or intravenous antimicrobial therapy especially against MDR bacteria or when remaining therapeutic options are limited (8, 12). Susceptibility testing for FO is difficult and requires AD as the reference method. Other methods, such as disk diffusion testing, only exist for certain indications or species (6, 19). For routine diagnostics, however, a ready to use, easy to store, and easy to handle test is needed. The ETEST FO by bioMérieux could offer such a solution.

In this study, the newly developed ETEST FO met the FDA performance criteria for testing of E. coli and E. faecalis. EA and CA above 90% were achieved, with no VME and only one ME. However, a trend to underestimate MIC values by the ETEST FO was observed especially for E. coli. The MIC values tend to be one or two doubling dilutions lower compared to the reference agar dilution method, this trend was especially observed at very low MIC values (±1 doubling dilution around 0.125 μg/mL). At those concentrations it has no consequences for interpreting low MIC values, but this observation explains the lower EA of 89.2% for the E. coli in challenge substudy. Therefore, a footnote stating this trend was added to the product labeling. Due to the same trending in one-doubling dilution steps, there was a high VME rate in clinical E. coli of 12.5% with EUCAST breakpoints for i.v. administration (breakpoint 32 μg/mL, Table 2), because all VME fell around the breakpoint of 32 μg/mL and were one dilution lower than reference testing. We must point out that if critical to patient care, testing should be repeated using an alternative or the reference method, prior to reporting results for E. coli, when the ETEST FO MIC is 32 μg/mL. The difference in VME rate between EUCAST and CLSI breakpoints can be explained because EUCAST does not offer an intermediate category.

The other ISO performance criteria with EA and CA > 90%, respectively, were met for E. coli (i.v. and oral application) and Enterobacterales (*n* = 305 clinical isolates). It must be noted that Enterobacterales did not meet VME criteria (≤3.0%) with three VME (8.8% 3/34) and three ME (1.1%), but the discrepancies between ETEST FO and AD were again only one doubling dilution apart; thus, the MIC values were within EA. The VME and ME were all caused by E. coli strains. At the time of testing, the only available fosfomycin resistant strains were E. coli (for numbers see Table 8) and 10strains for K. pneumoniae.

Trending to underestimate MICs was also observed with Citrobacter spp. and S. enterica. In contrast, K. pneumoniae MIC values tended to be at least one doubling dilution higher. Such a trend was not observed for E. faecalis.

Although the VME rate of Enterobacterales (8.8%) did not meet ISO requirements, the rate improved compared with the previous ETEST FM: A recent study (18) examined the ETEST FM MICs for Enterobacterales other than E. coli, and presented very high VME rates (32.1%, resistant isolates *n* = 53), especially for K. pneumoniae (75%, resistant isolates *n* = 16), K. aerogenes (50%, resistant isolates *n* = 4), and K. oxytoca/Raoutella spp. (50%, resistant isolates *n* = 1). Similar to Karlowsky et al., our results showed a low EA and a high VME rate for E. cloacae. The MIC values obtained with E. cloacae were generally higher when compared with E. coli and were mostly around the breakpoint of 32 μg/mL; thus, the ETEST FO lacks discrimination at this point. The performance criteria were not met for E. cloacae, and it is not recommended to use the ETEST FO for MIC testing of this species.

There are different EUCAST breakpoints for oral and i.v. breakpoints treatment because the breakpoints for uncomplicated urinary tract infections were reviewed late 2020 (4, 23) and led to the conclusion that a 90% reduction in growth of E. coli in urine is only achieved if the MIC was at a maximum of 8 μg/mL (5). The breakpoint was changed from 32 μg/mL to 8 μg/mL which might still be too high and the epidemiological cut-off value (ECOFF) is still under investigation (https://www.eucast.org/mic_and_zone_distributions_and_ecoffs/new_and_revised_ecoffs/). For i.v. administrations, there are limited data available and the breakpoint was not changed. It should be considered in the treatment, that bacteria with a MIC of >16 μg/mL very likely express some resistance mechanism, e.g., FosA (24).

Nevertheless, the number of resistant isolates for E. coli (see Table 8), K. pneumoniae (*n* = 10), and E. faecalis (*n* = 2) was low. At the time of testing there were no resistant isolates available for C. freundii, C. koseri, K. aerogenes, K. oxytoca, S. enterica, and S. marcescens. Therefore, the ability of ETEST FO to detect resistant isolates of these species is unknown.

Results for Staphylococcus spp. achieved good CA (98.0%; 98/100) and EA (94.0%; 94/100) with no VME. FO resistant isolates were also sparse with a total of eight strains.

Note that the reading of the S. aureus ETEST FO can be quite challenging, for clinical isolates as well as for the QC strain. That is because S. aureus isolates grow in a thin haze, that must be read. Macro- and microcolonies frequently occurred with Enterobacterales but did not hinder the reading and should not be considered for reading. E. faecalis shows no haze or colonies of any kind.

AST of FO is generally known to be difficult. Besides the fact that the reference method is AD, not broth microdilution, and that a supplement of glucose-6-phosphate is needed for testing, AD is only recommended for E. coli and E. faecalis (4, 19). The reading of test result, either of the ellipse or the zone diameter, is complicated by the manifestation of micro- and macrocolonies (25). In addition, EUCAST and CLSI have different approaches of how to interpret colonies in zone diameters. EUCAST favors to ignore colonies within a clear diameter, CLSI on the other hand offers no reading guide except M02 13th edition and M02QG 1st edition Volume 38, Number 4, January 2018 (6, 19). The appearance of colonies also applies for the newly developed ETEST FO and made reading of some of the MIC values difficult, especially for S. aureus. The user must be aware of the different reading instructions for different species in the package labeling.

In conclusion, results of this study support the efficiency of ETEST FO for determining MICs of many Enterobacterales, Staphylococcus spp., and E. faecalis, and may serve as an alternative to AD as reference method.
